# Spatially Resolved In Situ X‑ray Absorption Spectroscopy Studies of ZnS Nanoparticle Synthesis at the Water–Toluene Interface

**DOI:** 10.1021/acsnano.5c02875

**Published:** 2025-07-08

**Authors:** Lars Klemeyer, Francesco Caddeo, Tjark L. R. Gröne, Sani Y. Harouna-Mayer, Brian Jessen, Cecilia A. Zito, Jagadesh Kopula Kesavan, Ann-Christin Dippel, Fernando Igoa Saldaña, Olivier Mathon, Pieter Glatzel, Dorota Koziej

**Affiliations:** † Institute for Nanostructure and Solid-State Physics, Center for Hybrid Nanostructures, 14915 University of Hamburg, Luruper Chaussee 149, 22761 Hamburg, Germany; ‡ The Hamburg Center for Ultrafast Imaging, 22761 Hamburg, Germany; § Deutsches Elektronen-Synchrotron DESY, Notkestraße 85, 22607 Hamburg, Germany; ∥ ESRF, The European Synchrotron, 38043 Grenoble, France

**Keywords:** two-phase synthesis, HERFD-XANES, zinc sulfide, nanoparticle synthesis, nucleation and growth, focused X-ray spectroscopy, X-ray total scattering

## Abstract

Two-phase synthesis is a well-established approach for achieving precise control of the nanoparticle properties. However, studying and understanding chemical transformations in such a spatially heterogeneous system is challenging. In this work, we introduce a two-phase synthesis route for ZnS nanoparticles (ZnS NPs) at the water–toluene interface. By employing spatially resolved in situ high-energy resolution fluorescence-detected X-ray absorption spectroscopy (HERFD-XAS) combined with density functional theory (DFT) calculations, we track the diffusion of Zn^2+^ species at the interface, identify key reaction intermediates, and monitor the nucleation and growth of ZnS NPs within the toluene phase. We propose the formation of a [Zn­(H_2_O)_6_]^2+^ complex upon dissolving Zn­(Ac)_2_ in water and the diffusion of Zn^2+^ ions from water to toluene driven by the formation of an octahedral [Zn­(OA)_6_]^2+^ complex (OA = oleylamine). Furthermore, by complementing HERFD-XAS with total X-ray scattering analysis, we show the formation of an intermediate tetrahedral [Zn­(SR)_4_]^2+^ complex at 60 °C and its successive transformation to noncrystalline ZnS nuclei at 80 °C and crystalline ZnS NPs starting at 100 °C. Thus, we demonstrate how in situ X-ray spectroscopy can elucidate the coordination and diffusion of Zn^2+^ ions, and, in combination with X-ray scattering studies, identify the emergence of atomic and electronic structures during the two-phase synthesis of ZnS nanoparticles.

## Graphical abstract

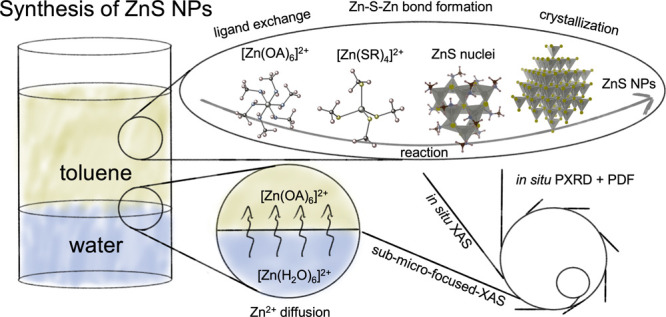

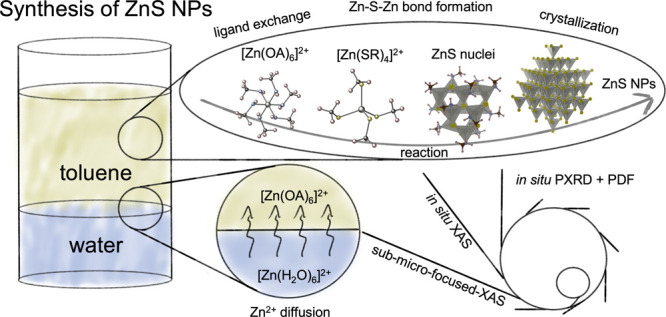

## Introduction

The synthesis of colloidal nanocrystals at the interface between two nonmiscible liquids has been the subject of intense investigation since the first report on thiol-functionalized Au nanoparticles synthesized at the water–toluene interface.^1^ The method has since been adapted and extended to the preparation of a number of metallic nanoparticles,^2–4^ alloys,^5^ various metal-oxides,^6–9^ and core–shell nanostructures.^10^

In a two-phase synthesis, molecular precursors are separated in the aqueous and organic phases, and nucleation and growth of nanocrystals are governed by diffusion processes occurring at the liquid–liquid interface.^11^ Compared with one-phase approaches such as hot-injection, the two-phase method offers precise morphological control and monodispersity using milder reaction conditions.^12^ Crucially, the two-phase method enables the use of common water-soluble metal precursors (e.g., acetates, nitrates, sulfates, etc.) while at the same time leveraging the benefits of organic-phase syntheses, such as employing capping agents to shape and stabilize the desired nanostructures into colloidally stable dispersions.^13^ Although the two-phase approach has been widely applied to synthesize various metal oxides, its use in preparing metal sulfides remains limited to a few reports focusing only on Cu_2_S,^14^ CdS^15,16^ and core–shell CdS–CdSe.^17^

Zinc sulfide (ZnS) is a wide bandgap semiconductor that has been intensively investigated as a promising candidate for a wide range of applications, including sensors,^18,19^ optoelectronics,^20,21^ photodetectors,^20^ solar cells,^22^ photocatalysis,^23,24^ and photoelectrochemistry.^25,26^ Colloidally stable ZnS nanostructures are commonly prepared via one-phase solvothermal^27,28^ or hot-injection routes^29^ using high boiling point organic solvents such as oleylamine or 1-octadecene. These methods have several drawbacks, including the requirement for very high reaction temperatures, inert atmospheres, and metal precursors that are soluble in nonpolar solvents (e.g., diethyl zinc), which are often difficult to synthesize and handle.

In this study, we propose a two-phase solvothermal method for preparing colloidally stable ZnS nanoparticles (ZnS NPs). The method involves the use of water-soluble zinc acetate (Zn­(Ac)_2_) as the metal precursor, while elemental sulfur and oleylamine are dissolved in the toluene phase. The reaction proceeds under mild conditions (100–155 °C) without requiring an inert atmosphere, yielding colloidally stable sphalerite ZnS NPs. We employ spatially resolved in situ high-energy-resolution fluorescence-detected X-ray absorption spectroscopy (HERFD-XAS) and in situ total X-ray scattering to monitor the synthesis. HERFD-XAS is an element-specific technique that provides detailed insights into the local atomic environment around the absorbing atom.^30–34^ The high-energy resolution mode enables us to discriminate between ZnS in sphalerite and wurtzite phases,^35^ where expected spectral differences between both phases are minor.^36^ In situ total scattering, particularly atomic pair distribution function (PDF) analysis, reveals information about interatomic distances present in the materials and, hence, local order, crystal structure, and domain size.^37–41^ By combining in situ HERFD-XAS with X-ray scattering,^42^ we track diffusion processes at the water–toluene interface, identify molecular intermediates, and monitor the nucleation and growth of ZnS NPs, as shown in Figure 1. We find that Zn^2+^ ions diffuse from the water to the toluene phase, driven by the formation of a stable octahedral [Zn­(OA)_6_]^2+^ complex (OA = oleylamine). We propose that a tetrahedral [Zn­(SR)_4_]^2+^ complex (R = undefined organic rest) forms during the reaction at 60 °C, which subsequently converts to ZnS nuclei at 80 °C, promoting the growth of crystalline sphalerite ZnS NPs starting at 100 °C.

**Figure 1:**
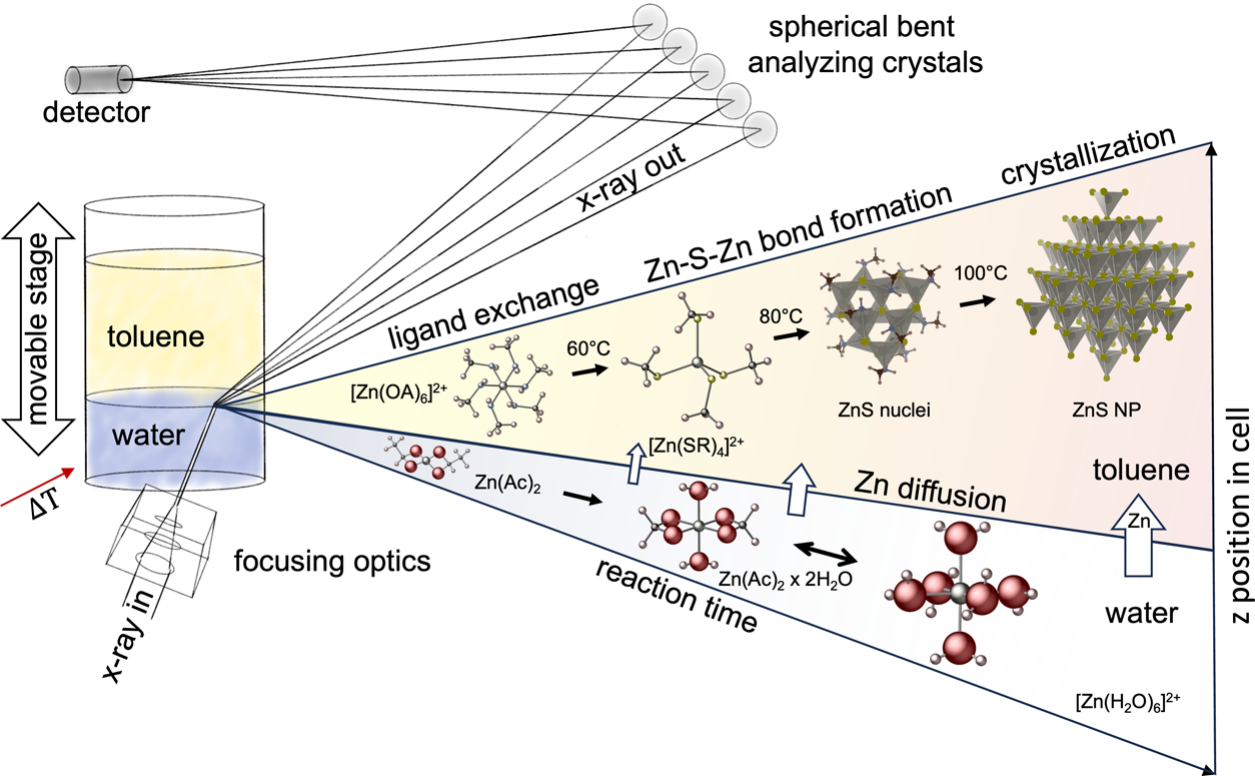
Schematic illustration of the in situ HERFD-XAS experiment to monitor the synthesis of ZnS NPs at the water–toluene interface and overview of the main findings. The spatial resolution provided by a movable stage and a microfocused beam enables one to probe separately the water and toluene phases. The Zn­(Ac)_2_ dissolved in water forms a mixture of [Zn­(Ac)_2_(H_2_O)_2_] and [Zn­(H_2_O)_6_]^2+^ complexes, where the Zn^2+^ ions are octahedrally coordinated with six water molecules. During the reaction, the Zn^2+^ ions diffuse into the toluene phase and form an octahedral [Zn­(OA)_6_]^2+^ complex. This complex converts at 60 °C to a tetrahedral [Zn­(SR)_4_]^2+^ complex, which then forms ZnS nuclei at 80 °C. The crystallization of sphalerite ZnS NPs starts at 100 °C.

## Results and Discussion

We prepare colloidally stable ZnS NPs adopting a two-phase water–toluene method, where zinc acetate (Zn­(Ac)_2_) is dissolved in water, and elemental sulfur and oleylamine are dissolved in toluene, acting as the sulfur source and ligand, respectively (see the Experimental Section for the synthesis details). The average size of sphalerite ZnS can be tuned from 2.3 to 7.5 nm by adjusting the temperature between 100 and 150 °C and reaction time, as indicated in SEM and PXRD analysis, shown in Figure SI1a–d.

To unravel the reaction steps leading to the formation of the ZnS NPs, including the diffusion of Zn species from water to the toluene phase, we carry out HERFD-XANES employing a microfocused beam in combination with a movable stage, which allows us to discriminate between both phases, with a spatial resolution limited by the beam size of 0.5 × 0.5 μm.^43^ We carry out the reaction employing an in situ cell described in a previous work.^35^

The sphalerite ZnS NPs synthesized in the in situ cell at 155 °C for 1h have a size distribution close to that obtained in a conventional autoclave under similar reaction conditions, as shown in Figures SI2–S4.

We monitor the diffusion of Zn species at the water–toluene phase, collecting HERFD-XAS data using a micrometer-focused beam at room temperature, following the contact of the two phases. Figure 2a,b shows the XAS spectra collected at room temperature in the water and toluene phases, respectively, alongside simulated XAS spectra of the expected complexes obtained via ORCA DFT calculations.^44^ We observe that the white line intensity in the experimental XANES spectrum of Zn­(Ac)_2_ increases upon dissolution, while the *E*_0_ position remains largely unchanged. This suggests an increase in coordination number from 4 (tetrahedral) to 6 (octahedral). A similar trend is observed in the simulated spectra, where Zn­(Ac)_2_ and the octahedral [Zn­(H_2_O)_6_]^2+^ complex exhibit a comparable change in white line intensity. Moreover, the position of the white line also matches the presence of a [Zn­(H_2_O)_6_]^2+^ complex. We therefore propose that upon dissolution of the Zn­(Ac)_2_ used as a precursor, the Zn^2+^ ion forms the octahedral [Zn­(H_2_O)_6_]^2+^ complex with six water molecules, as shown in the inset in Figure 2a. The broadening of the white line in Figure 2a suggests that Zn­(Ac)_2_ and [Zn­(Ac)_2_(H_2_O)_2_] might still be present in the water phase following reaction 1. The formation of a [Zn­(Ac)_2_(H_2_O)_2_] complex during the dissolution of Zn­(Ac)_2_ in water is described in the literature.^45^

$$\mathrm{Zn}(\mathrm{Ac}{)}_{2}\overset{+2H_{2}O}{\to }[\mathrm{Zn}(\mathrm{Ac}{)}_{2}(H_{2}O{)}_{2}]⇌[\mathrm{Zn}(H_{2}O{)}_{6}{]}^{2+}+2{\mathrm{Ac}}^{-}$$ 1

When moving to the toluene phase, we record a Δ*E*_0_ shift of 1.3 eV, visible in Figure 2e, which is compatible with a change from a Zn–O to a Zn–N coordination,^35^ likely due to the coordination of Zn^2+^ by oleylamine molecules present in toluene. The comparison of the experimental white line shape with different Zn-oleylamine complexes simulated by the ORCA calculations, Figure 2b, points to the presence of a [Zn­(OA)_6_]^2+^ (OA = oleylamine) complex in the toluene phase, where Zn^2+^ ions are octahedrally coordinated by six oleylamine molecules. However, all the ORCA simulated spectra show discrepancies with the experimental spectra since ORCA fails to reproduce the exact line shape of experimental XANES. This is already observed for Zn-complexes in the literature.^33,35^ Nevertheless, by comparing spectral shape, white line intensity, and *E*_0_ between experimental and simulated spectra, we confidently identify the presence of [Zn­(H_2_O)_6_]^2+^ and [Zn­(OA)_6_]^2+^complexes as the predominant species in water and toluene, respectively.

**Figure 2:**
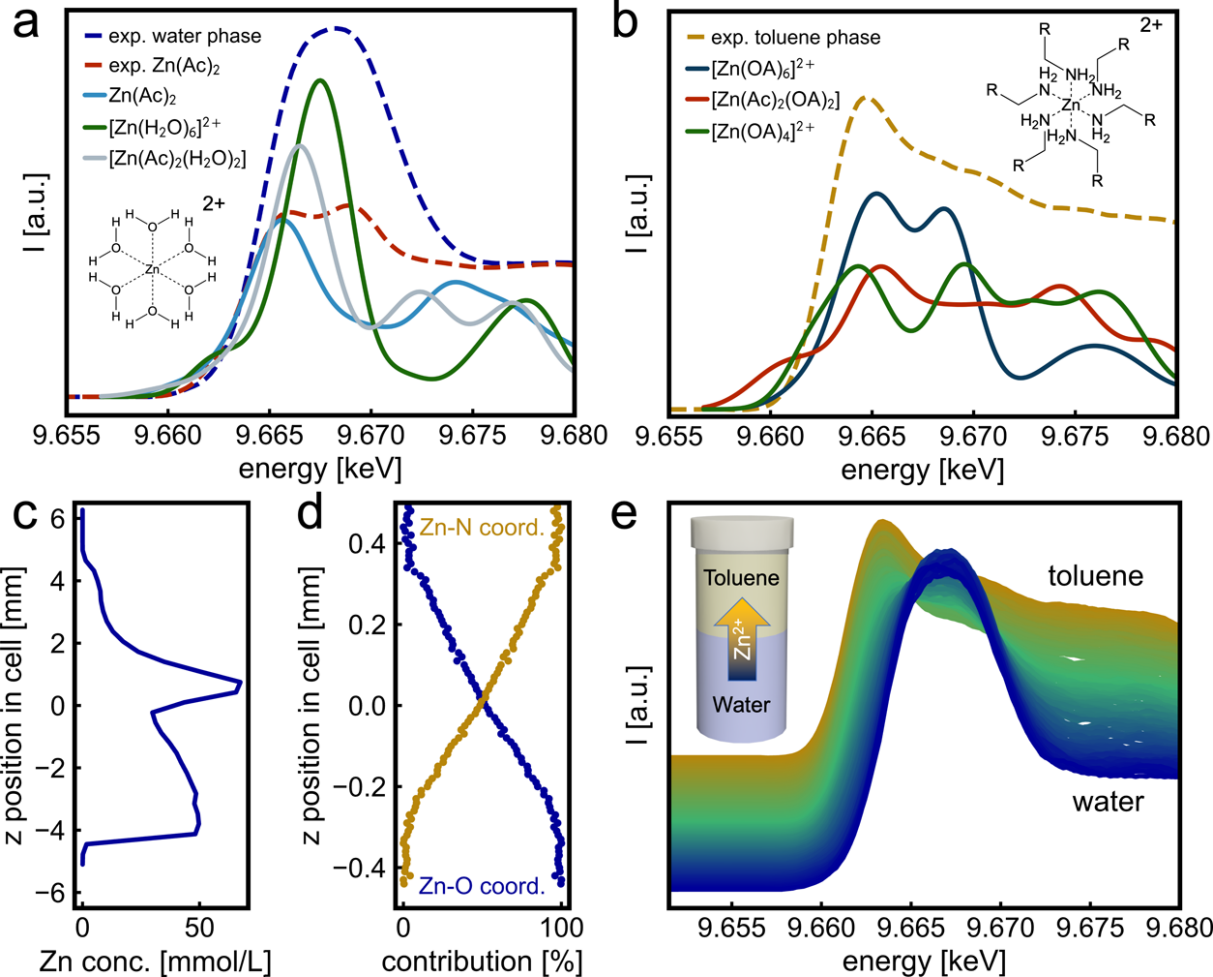
Diffusion of Zn^2+^ species at room temperature at the water–toluene interface monitored by HERFD-XAS. XAS spectra at the Zn K-edge in the water (a) and toluene (b) phases, compared with an experimental Zn­(Ac)_2_ reference and with the calculated XAS spectra by DFT calculations of [Zn­(H2O)_6_]^2+^, Zn­(Ac)_2_, [Zn­(Ac)_2_(H2O)_2_]^2+^ and [Zn­(OA)_6_]^2+^, [Zn­(Ac)_2_(OA)_2_], and [Zn­(OA)_4_]^2+^, respectively. The concentration profile of Zn at the water–toluene interface (c) and the fraction profile of [Zn­(H_2_O)_6_]^2+^ and [Zn­(OA)_6_]^2+^ were extracted by MCR-ALS analysis of the HERFD-XAS spectra (d). The HERFD-XAS spectra were recorded at different positions z in the cell at RT. The inset shows a schematic drawing of Zn^2+^ diffusion at room temperature across the water–toluene interface (e).

To quantify the distribution of the Zn^2+^ species at room temperature, we collected HERFD-XAS spectra scanning across the water–toluene interface. We first observe a gradual energy shift of the *E*_0_ by 1.3 eV toward lower energy, Figure 2e, as expected for a change in the local environment around the Zn atoms from a Zn–O to a Zn–N coordination.^35^ The *E*_0_ corresponds to the energy value where the first derivative of the XAS reaches its maximum, while the white line is the maximum intensity in the XAS spectra.^35,46,47^

Scanning the water–toluene interface while collecting the intensity of the K_α1_ emission under illumination at 10 keV allows for the extraction of the concentration profile of the Zn species across the water–toluene interface, as shown in Figure 2c. Moving from the water to the toluene phase, the Zn concentration decreases gradually when approaching the interface at the z position 0, shows a peak slightly above the interface on the toluene side, and then further decreases moving away from the interface. This agrees with a diffusion process across the water–toluene interface, likely driven by the formation of a stable [Zn­(OA)_6_]^2+^ complex in toluene.

The collected HERFD-XAS data allow us to determine the fraction of [Zn­(H_2_O)_6_]^2+^ [Zn­(OA)_6_]^2+^ complexes around the interface, Figure 2d, as recovered by multivariate curve resolution-alternating least squares (MCR-ALS) analysis of the spatially resolved HERFD-XAS data, shown in Figure 2e.^48,49^ More details about the MCR-ALS analysis are available in Figures SI5–SI7 and Tables SI1, SI2. The analysis confirms that at room temperature, i.e., before the start of the reactions leading to the formation of the ZnS NPs, the Zn ions are distributed across the water–toluene interface through a diffusion process, with the formation of a [Zn­(OA)_6_]^2+^ complex in toluene occurring at the expenses of the [Zn­(H_2_O)_6_]^2+^ complex present in water. To exclude beam damage in the XAS measurements, extensive beam damage studies were performed in both phases, as shown in Figure SI8.

During the formation of ZnS NPs, one can observe color changes in the water and toluene phases, as shown in Figure 3a. The Zn^2+^ concentration profiles before (blue curve) and after the reaction (red curve) show that a large fraction of Zn^2+^ is still dissolved at room temperature in the water phase. During the reaction, with an increasing temperature, the Zn^2+^ diffuses from the water to the toluene phase up to around 80 °C, when the diffusion is complete. Therefore, we assume that the diffusion process does not limit the nucleation or the growth rate of ZnS NPs, which occurs at temperatures higher than 80 °C. The evolution of the Zn^2+^ content in the toluene phase during the reaction is shown in Figure SI9.

**Figure 3:**
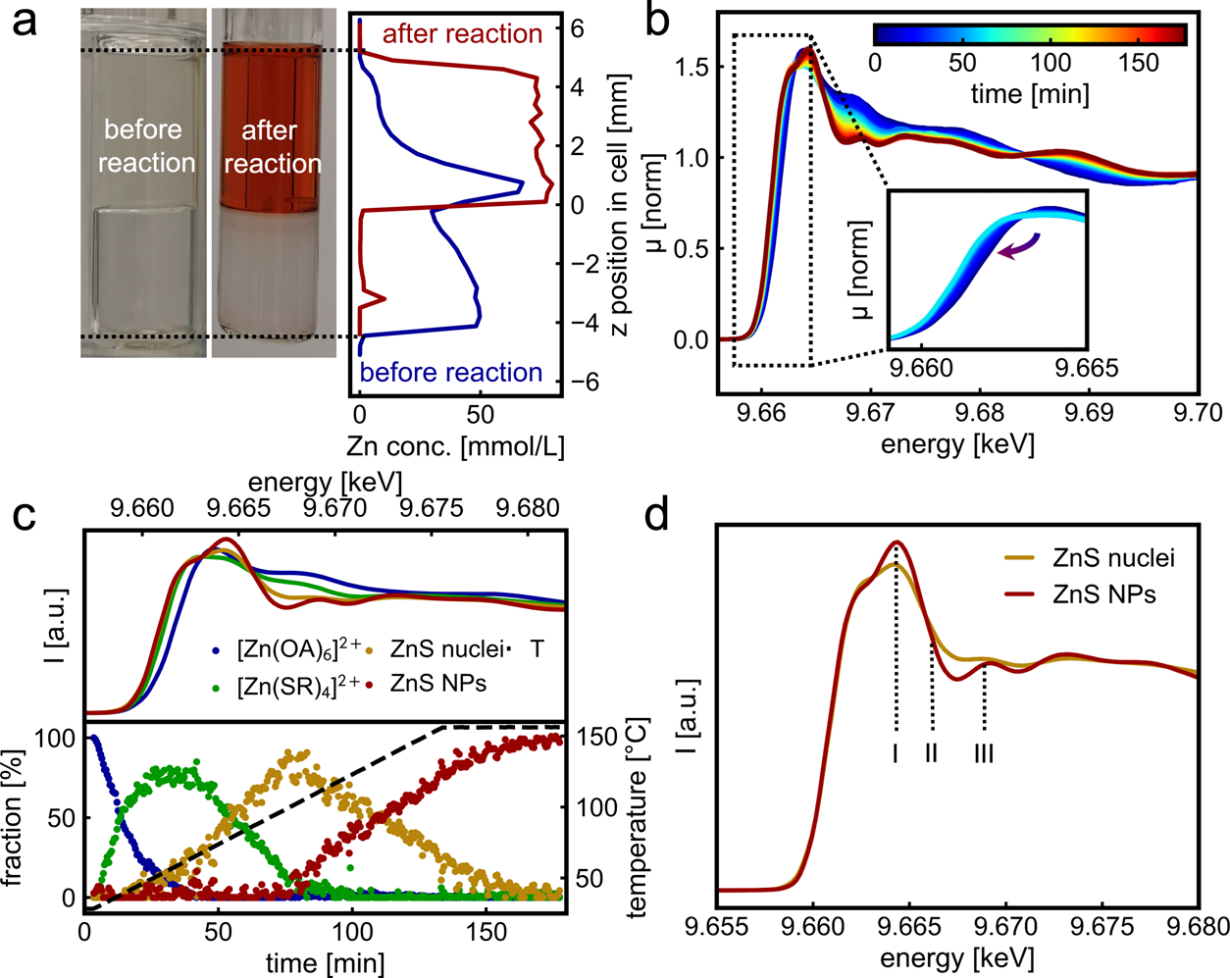
Diffusion of Zn complexes, ligand exchange, and nucleation of ZnS investigated by in situ HERFD-XAS and MCR-ALS analysis. Photographs of reaction solutions (a) prior (left) and after the reaction (middle) and the corresponding Zn concentration profiles around the interface (right). In situ HERFD-XAS data for (b) reveal the transformation of [Zn­(OA)_6_]^2+^ into ZnS NPs. MCR-ALS analysis (c) deconvolutes the in situ HERFD-XAS data into four distinct components (top) and their related concentrations (bottom). Comparison of the XAS spectra (d) of the second intermediate (yellow) and the final product (red) obtained from MCR-ALS analysis.

To monitor the reaction pathways leading to the formation of ZnS NPs in the toluene phase, we recorded in situ HERFD-XAS at different positions in the toluene phase. During the experiment, the reaction is performed with a slow heating rate of 1 °C/min, allowing for monitoring of the presence of reaction intermediates. The in situ HERFD-XAS data (Figure 3b) reveal an *E*_0_ shift by around 1 eV to lower energies when the reaction temperature increases to 60 °C (first 40 min). This energy shift suggests a change in the coordination environment around Zn, where the amine ligand (Zn–N coordination) is likely substituted by thio-ligands (Zn–S),^50,51^ as highlighted in the inset, and discussed in Figure SI10. The line shape of HERFD-XAS at 60 °C differs from the one expected for ZnS, as shown in Figure SI11, which highlights the occurrence of reaction intermediates.

We therefore perform MCR-ALS analysis of the HERFD-XAS data set, as shown in Figure 3c. MCR-ALS can deconvolute the observed changes in the in situ HERFD-XAS data into four individual contributions. Figure 3c shows the XAS spectra of each contribution (top) extracted from the MCR-ALS analysis and their related concentration profiles during the reaction (bottom panel). The starting point of the reaction (blue line) is attributed to the [Zn­(OA)_6_]^2+^ complex described in Figure 2b. The XAS spectra of the first intermediate (green line) show, in addition to the *E*_0_ shift of around 1 eV that is characteristic of a Zn–S coordination, a slight decrease in white line intensity compared to the [Zn­(OA)_6_]^2+^ complex, as highlighted in the inset of Figure 3b. The decrease in white line intensity might indicate a change in coordination geometry, potentially from octahedral to tetrahedral.^33,52^ In Figure SI12, we compare the XAS spectra of various tetrahedral and octahedral Zn–S complexes simulated by ORCA DFT with the XAS spectra of the intermediate extracted from MCR-ALS analysis (green line). We find that the best-fitting simulated spectra correspond to a [Zn­(oleylthioamide)_4_]^2+^ ([Zn­(SOA)_4_]^2+^) complex. The simulated and experimental spectra match well in terms of white-line and intensity, with some discrepancies in the postedge features originating from the second coordination sphere around Zn. In an earlier study, we performed DFT calculations of various tetrahedral Zn–S complexes, and we found that a tetrahedral [Zn­(SOA)_4_]^2+^ complex forms also when zinc acetate is dissolved in oleylamine with the presence of elemental sulfur.^35^ This complex likely occurs as an intermediate during our two-phase reaction in the toluene phase due to the presence of oleylamine and sulfur. However, due to the aforementioned discrepancies in the postedge features of the XAS spectra, we assign this first intermediate to a generic tetrahedral [Zn­(SR)_4_]^2+^ complex, where R = to an undefined organic group. Based on the literature, we assume that the SR group may correspond to an oleylthioamide group,^35,53^ which we were unable to confirm with the performed analysis. The formation of molecular Zn–S complexes during the one-pot synthesis of ZnS NPs has already been proposed in the literature.^35,54–56^

A detailed analysis of individual XAS features is required to identify the second intermediate recovered by MCR-ALS. Often, the formation of ZnS NPs in the sphalerite phase proceeds via an intermediate in the wurtzite phase,^35^ which only differs in the stacking arrangement of the ZnS tetrahedra. We excluded the presence of wurtzite ZnS during the reaction by FDMNES calculations, shown in Figure SI13a and by PXRD analysis in Figure 4e.^31,36,57^ Nevertheless, the less pronounced features in the postedge region of the ZnS nuclei (yellow), as shown in Figure 3d, suggest a lack of periodicity in their atomic structure compared to the ZnS NPs (red).^58^ The experimental spectra of ZnS NPs were compared with those of a ZnS reference in Figures SI13 and S14.

**Figure 4:**
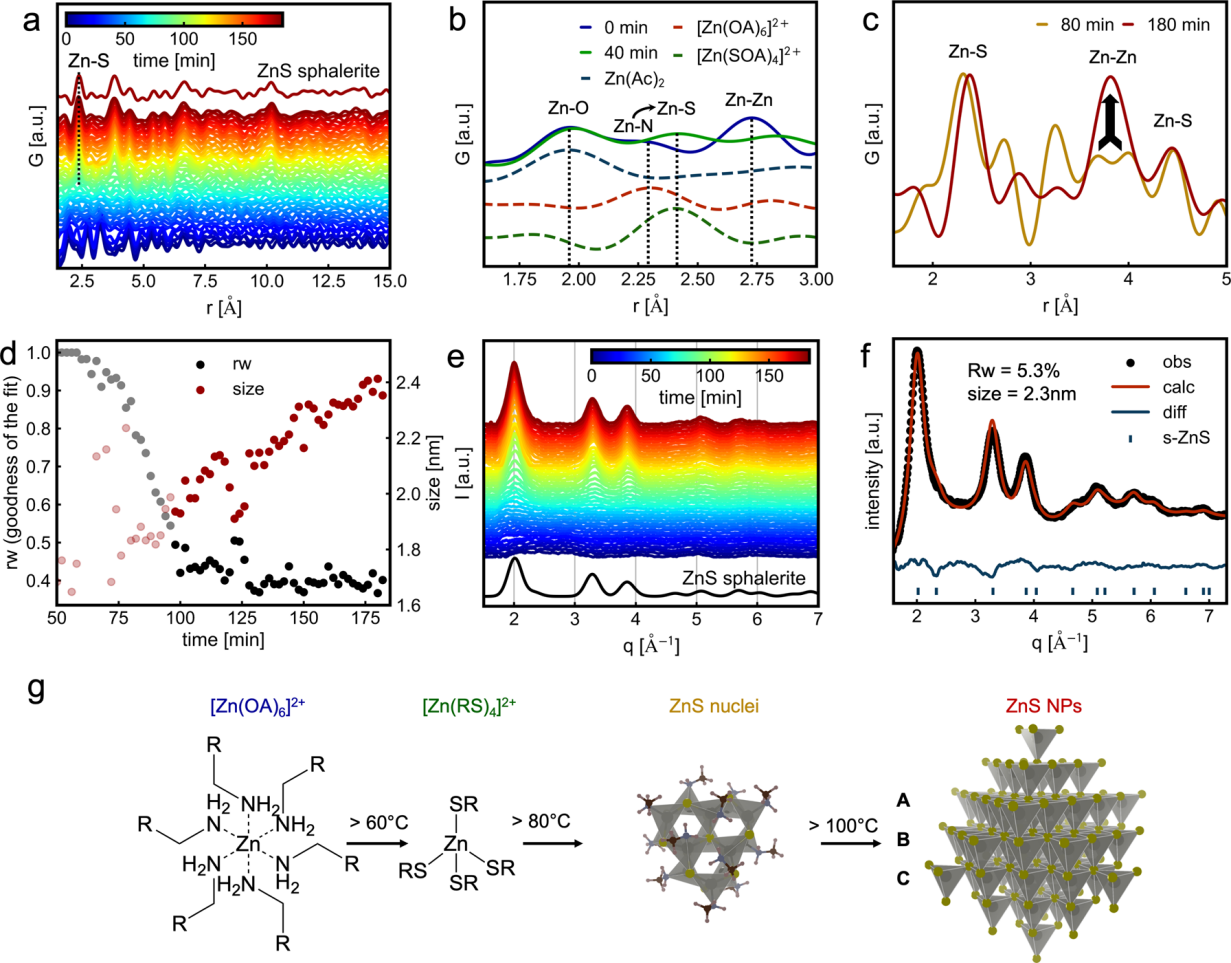
Ligand exchange, nucleation, crystallization, and growth investigated by in situ PDF and PXRD analysis. In situ PDF analysis (a) showing the emergence of Zn–S interatomic distances (dotted line) after 30 min reaction time. The comparison of the PDF (b) at the beginning of the reaction (solid blue) and after 40 min of reaction time (solid green), with simulated PDFs of Zn­(Ac)_2_ (dashed blue), [Zn­(OA)_6_]^2+^ (dashed red), and [Zn­(SOA)_4_]^2+^ (dashed green). The experimental PDFs are normalized to the Zn–O peak. Comparison of PDF (c) at 80 min (100 °C) and 180 min reaction time, reflecting the PDF of ZnS nuclei (yellow) and ZnS NPs (red). Both PDFs were normalized to the Zn–S peak height. The in situ PDF fitting of (d) with ZnS in sphalerite phase. The fit becomes meaningful after a 100 min reaction time (120 °C) when the rw value drops below 0.5, visualized by blurring the markers before 100 min reaction time. In situ PXRD analysis (e) shows the emergence of ZnS in the sphalerite phase (s-ZnS) during the reaction without any ZnS content in the wurtzite phase. Rietveld refinement (f) of the final PXRD pattern confirms a domain size of around 2.3 nm in the ZnS NPs. The proposed reaction mechanism is illustrated in (g).

To resolve the atomic structure of the second intermediate and monitor nucleation and growth of the ZnS NPs during synthesis, we perform in situ total scattering (TS) measurements at the water–toluene interface and calculate the atomic-pair distribution function (PDF) to achieve a histogram of interatomic distances, as shown in Figure 4a. The dashed line highlights the emergence of the Zn–S interatomic distance, starting above 60 °C. This aligns with the presence of the [Zn­(SR)_4_]^2+^ complex, evidenced by MCR-ALS analysis of the in situ XAS data. In contrast to the XAS analysis, the TS data were collected with a 1 mm^2^ beam size, with which we probed the water and toluene phases simultaneously.

Since we perform the TS experiment and the XAS experiment under similar reaction conditions, we assume that the in situ reaction follows the same reaction kinetics. As already described, the Zn^2+^ content diffuses into the toluene phase. Due to the high atomic number of Zn, in comparison to the other chemical species in solution, the diffraction signal in the TS data will be dominated by the Zn content. As shown in Figure SI9, the diffusion process reached equilibrium at around 50 min of reaction time, where the majority of Zn^2+^ diffuses into the toluene phase. Therefore, we assume that we observe TS signal from Zn species in the water and toluene phase at 0 min ([Zn­(H_2_O)_6_]^2+^ and [Zn­(OA)_6_]^2+^), and 40 min ([Zn­(H_2_O)_6_]^2+^ and [Zn­(SR)_4_]^2+^), and that we observe the TS signal mainly from Zn species located in the toluene phase at 80 min (ZnS nuclei) or at the end of the reaction (ZnS NPs), as shown in Figure 4b,c. At the beginning of the reaction, the PDF (blue) corresponds to a mixture of zinc complexes in both water and toluene, as shown by the presence of Zn–O and Zn–N interatomic distances. Furthermore, the [Zn­(H_2_O)_6_]^2+^ complex in the water phase might partly self-assemble into a molecular cluster as indicated by an increased extent of correlations and larger peak heights in the PDF, as discussed in Figure SI15 and observed for other transition-metal acetates in water.^59,60^ The decrease in the PDF peak amplitudes during the first 30 min, Figure 4a, can be attributed to the diffusion of Zn^2+^ to the toluene phase away from the interface, which lowers the Zn concentration in the water phase and, consequently, in the beam path. After reaching 60 °C (40 min into the reaction), the Zn–N peak disappears, substituted by an established Zn–S coordination, shown in Figure 4b (green line), characteristic of the [Zn­(SR)_4_]^2+^ complex. At around 100 °C (80 min into the reaction), the PDF corresponding to the second intermediate, Figure 4c (yellow line), shows delocalized Zn–Zn interatomic distances at 3.3, 3.7, and 4.0 Å, besides the Zn–S interatomic distance at 2.4 Å, which do not correspond to the presence of wurtzite ZnS NPs. Additionally, the extent of the correlation in the PDF of the second intermediate is limited to around 10 Å, as highlighted in Figure SI16. Therefore, we propose that the second intermediate corresponds to small ZnS nuclei whose atomic arrangements do not have long-range ordering. The presence of such ZnS nuclei as an intermediate has already been discussed in the literature for other ZnS synthesis routes.^61,62^

After the reaction temperature is increased above 100 °C, the ZnS nuclei evolve into crystalline ZnS NPs, as evidenced by the appearance of a strong peak at 3.8 Å corresponding to the typical Zn–Zn distance in sphalerite, Figure 4c. The ZnS NPs show a continuously increasing extent of correlations, reflecting their growth. The corresponding refinement is shown in Figure 4d. The refined PDF of ZnS in the sphalerite phase is compared to the last experimental PDF of the in situ run in Figure SI17. The domain size of the ZnS NPs grows up to 2.3 nm for a reaction time of 1 h. Details about the PDF fitting procedure are shown in Figure SI18.

In situ powder X-ray diffraction analysis (Figure 4e) reveals the crystallization of ZnS NPs in the sphalerite phase starting at 100 °C and does not provide any evidence of the presence of an intermediate wurtzite ZnS phase, in line with HERFD-XAS and PDF analysis. Consequently, we can rule out the formation of wurtzite ZnS NPs, which is instead observed in other ZnS syntheses.^35,54–56^ Additionally, the ZnS nuclei, which form during the reaction at around 80 °C, do not exhibit a long-range order since a long-range order in the ZnS nuclei would result in visible contributions in the PXRD pattern. Therefore, we suggest that nanoparticle growth occurs above 100 °C, where ZnS nuclei grow directly into crystalline ZnS sphalerite particles. The PXRD pattern of the resulting ZnS NPs is further analyzed by Rietveld refinement and confirms the domain size of around 2.3 nm, as shown in Figure 4f.

By integrating information on Zn coordination throughout the synthesis from in situ HERFD-XAS data analysis with interatomic distances and sizes obtained from in situ PDF analysis, along with insights into the crystallographic phase from in situ PXRD data, we propose a reaction mechanism for the two-phase synthesis of ZnS NPs, as illustrated in Figure 4g. The oleylamine ligands in the [Zn­(OA)_6_]^2+^ complex in toluene are replaced by sulfur ligands at temperatures above 60 °C, forming a [Zn­(SR)_4_]^2+^ complex. Reaching around 80 °C, the ZnS tetrahedra undergo Zn–S–Zn bond formation, arranging into a nonperiodic structure and creating noncrystalline ZnS nuclei approximately 10 Å in size. The crystallization of ZnS NPs in the sphalerite phase starts at 100 °C, followed by continuous domain growth, reaching approximately 2.3 nm after 60 min at 155 °C.

## Conclusions

In this work, we present a synthetic route yielding sphalerite ZnS NPs at the water–toluene interface. We provide spatially resolved insights into the coordination chemistry and structural changes occurring during the synthesis of ZnS NPs in a two-phase system. The in situ HERFD-XANES showed that Zn^2+^ species diffuse from water to toluene through an equilibrium involving [Zn­(H_2_O)_6_]^2+^ and [Zn­(OA)_6_]^2+^ complexes forming in water and in toluene, respectively. The in situ studies reveal at 60 °C a ligand exchange process where the octahedral [Zn­(OA)_6_]^2+^ complex evolves into a tetrahedral [Zn­(SR)_4_]^2+^ complex. Above 80 °C, this complex subsequently condenses to form Zn–S–Zn bonds, leading to ZnS nuclei, which then grow into crystalline sphalerite ZnS NPs above 100 °C. The studies are complemented by in situ X-ray scattering measurements, which further confirm the transformation of noncrystalline ZnS nuclei directly to ZnS in the sphalerite phase.

In conclusion, this work highlights the potential of spatially resolved HERFD-XANES as a tool for monitoring chemical reactions in nonhomogeneous two-phase reactions that are inaccessible to other methodologies. The insights gained through this approach not only enhance our understanding of two-phase ZnS synthesis but also open new opportunities for exploring the synthesis of a wider range of nanostructures or quantum dots.

## Experimental Section

### Chemicals

Zinc­(II) acetate (Zn­(Ac)_2_) (99.99%, anhydrous), sulfur (99.998% trace metal basis), toluene (anhydrous, 99.8%), and oleylamine (≥98% primary amine) were procured from Sigma-Aldrich. All chemicals were utilized in their as-received states without undergoing additional purification.

### ZnS Synthesis

For the preparation of ZnS precursor solutions, 0.184 g of Zn­(Ac)_2_ (1 mmol) was dissolved in 15 mL of Milli-Q water. The resulting solution was subjected to filtration by using 200 nm screw-on syringe filters. Simultaneously, 1.5 mL of oleylamine (∼98%) and 0.096 g (3 mmol) of elemental sulfur were dissolved in 15 mL of anhydrous toluene. For the in situ studies, 50 μL of the aqueous solution and 100 μL of the toluene solution were sequentially introduced into the reaction cell, and the reaction was performed with a heating rate of 1 °C/min up to 155 °C for 1 h. A control reaction was also carried out in a standard 45 mL stainless-steel autoclave, with a temperature range of 100–150 °C and reaction times from 1 to 24 h.

### Beamline Setup and Data Acquisition

The HERFD-XANES were acquired at the ID26 and ID24 beamlines at the European Synchrotron Radiation Facility (ESRF) in Grenoble, France. On both beamlines, the Si(111) reflection of a double crystal monochromator was chosen for selecting the incoming energy. The HERFD-XANES data were obtained by measuring the intensity of the Zn Kα main line utilizing Si(642) crystals in Rowland Geometry within a Johann-type X-ray emission spectrometer.^65^ The crystal bending radius was 1 m on ID26 and 0.5 m on ID24. This measurement involved continuous scanning of the monochromator, together with the undulator gap. The scans across the interface shown in Figure 2 were performed at ID24, with a focused beam size of 0.5 μm × 0.5 μm. The in situ data shown in Figure 3 were collected at ID26 with a beam size of 0.2 × 0.4 mm. The XANES spectra of the ZnS reference in HERFD-XAS mode were compared with conventional transmission XAS in Figure SI19. To minimize radiation damage, the position of the X-ray beam center was varied among 12 different points on the reaction cell, with three positions in the horizontal direction and four positions in the vertical direction. XANES spectra were recorded at intervals of 16 s, with the energy range set from 9.64 to 9.8 keV and an energy step of 0.2 eV.

The in situ X-ray total scattering data were obtained at the P21 beamline of PETRA III, situated at the Deutsches Elektronen-Synchrotron (DESY) in Hamburg, Germany.^66^ The total scattering measurements were performed at intervals of 0.5 s using a flat panel detector (PerkinElmer XRD1621, Varex Imaging Corp.) featuring 2048 × 2048 pixels with a size of 200 × 200 μm^2^. Throughout the experiments involving the synthesis of ZnS NPs at 155 °C, the sample-to-detector distance (SDD) for the total scattering data was set at 0.39 m. The SSD for the XRD data was set to 1.54 m. These distances were determined through calibration with a LaB6 calibrator, and the X-ray beam energy employed for the measurements was 101.8 keV.

### Data Processing

The HERFD-XAS were processed through a custom Python code built upon previously published packages. Edge position determination and edge jump normalization were executed using the LARCH-XAFS module.^67^ Spectroscopic data underwent treatment with a Savitzky-Golay filter and further processing utilizing the NumPy^68^ and SciPy^69^ packages. XANES spectra simulations were carried out using ORCA 5.0.4. code,^70^ where the initial complex before relaxation was built with Avogadro 1.2.0, an open-source molecular builder and visualization tool.^71^ XANES spectra simulations in the SI were conducted using the FDMNES code.^72^ ORCA and FDMNES parameters are listed in the SI.

Azimuthal and radial integration of the 2D detector patterns for PDF and XRD data was done using the Python module pyFAI,^73^ excluding beam stop shadows, glitches, defective, and noisy pixels. Background data were collected under identical reaction conditions of the ZnS syntheses at 155 °C. The background data were subtracted from the sample data set using a fitted scaling factor constrained to a maximum value between 0.95 and 1.1. Data averaging over 120 frames, corresponding to a 1 min time resolution, was performed. PDFs (*G*(*r*)) were calculated in the *r*-range (lowest to highest *r* extracted from the Fourier transformation) of 0–30 Å^–1^ in steps of 0.01 Å^–1^ using the software PDFgetX3.^74^ The PDFs were obtained with a *Q*_maxinst_ (highest *Q* usable under given experiment conditions) of 25 Å^–1^ over a *Q*-range (upper to lower cutoff) of 0.1–14.8 Å^–1^ and an *r*-poly of 1.1. The latter corresponds to the range of polynomial fitting in ad hoc correction (here, a five ° polynomial correction, *n* = *r-*poly*Q*_maxinst_/π). The PDF fittings were performed by utilizing Diffpy-CMI, employing a single-phase refinement with ZnS sphalerite. The sphalerite phase (mp-10695) was extracted from the Materials Project database,^75^ and the refinement was conducted sequentially, starting from the PDF at the end of the reaction and proceeding backward to earlier reaction times to ensure improved fit reliability. The Rietveld refinement was performed with the GSAS-II package.^76^ The sphalerite phase (ICSD-230703) was used from the ICSD database. The refinement was carried out in a sequential way, starting from XRD at the end of the reaction and going backward to earlier reaction times, ensuring a better reliability of the fit.

The wording and readability of the manuscript were improved by ChatGPT 4.0, following the guidelines of ACS Journals.^77^

## Supplementary Material



## Notes

TEM and HRTEM analysis, DFT calculations, MCR-ALS fitting parameters, radiation damage study, Zn concentration profile during the reaction, FDMNES calculations, reference spectra, and PDF analysis (PDF)
