# Supplementary material for: Spatially Resolved In Situ X‑ray Absorption Spectroscopy Studies of ZnS Nanoparticle Synthesis at the Water–Toluene Interface
Source: ACS Nano. 2025 Jul 8;19(28):25710–9. doi: 10.1021/acsnano.5c02875 (PMC12291586; doi:10.1021/acsnano.5c02875)
Supplement: Supplementary file 1 [file nn5c02875_si_001.pdf]

Supporting Information:
Spatially Resolved *in situ* X-ray Absorption Spectroscopy Studies of ZnS Nanoparticle Synthesis at the Water-Toluene Interface

Lars Klemeyer[1,2,†], Francesco Caddeo[1,†], Tjark L. R. Gröne[1], Sani Y. Harouna-Mayer[1,2], Brian Jessen[1], Cecilia A. Zito[1,2], Jagadesh Kopula Kesavan[1,2], Ann-Christin Dippel[3], Fernando Igoa Saldaña[3], Olivier Mathon[4], Pieter Glatzel[4], and Dorota Koziej[1,2]*

[1] University of Hamburg, Institute for Nanostructure and Solid-State Physics, Center for Hybrid Nanostructures, Luruper Chaussee 149, 22761 Hamburg, Germany

[2] The Hamburg Center for Ultrafast Imaging, 22761 Hamburg, Germany

[3] Deutsches Elektronen-Synchrotron DESY, Notkestraße 85, 22607 Hamburg, Germany.

[4] ESRF, The European Synchrotron, 38043 Grenoble, France

† These authors contributed equally

[*] Corresponding author

TEM analysis of ZnS NPs produced in the autoclave:

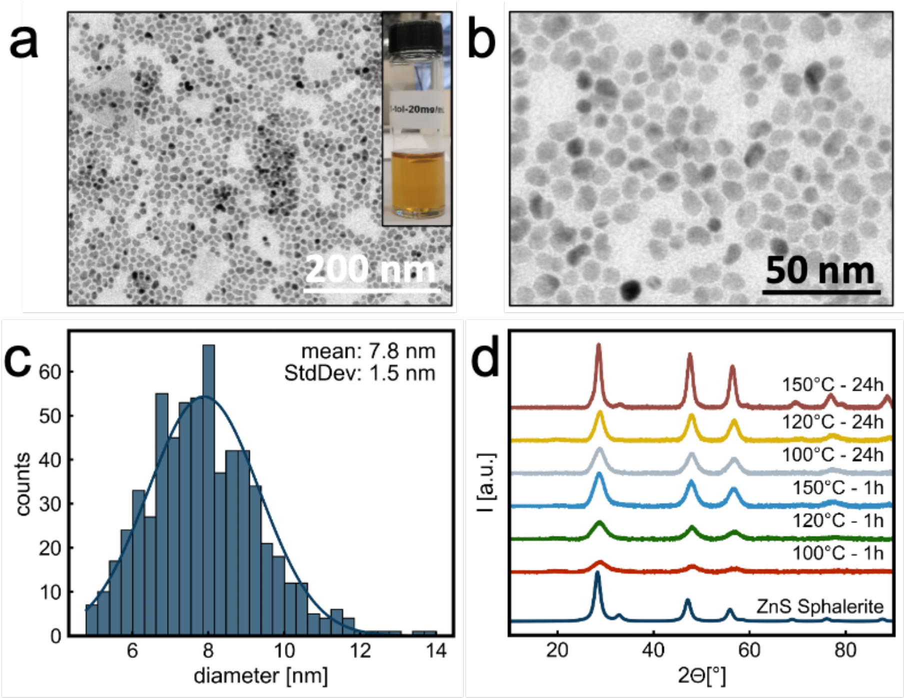

Figure SI1: TEM images (**a**,**b**) and particle size distribution (**c**) of ZnS NPs produced in an autoclave at 150 °C for 24 hours; XRD patterns (**d**) of ZnS NP produced in an autoclave with reaction temperatures between 100 and 150 °C and reaction time of 1 h and 24 h.

TEM and HRTEM analysis of ZnS NPs produced in the *in situ* cell:

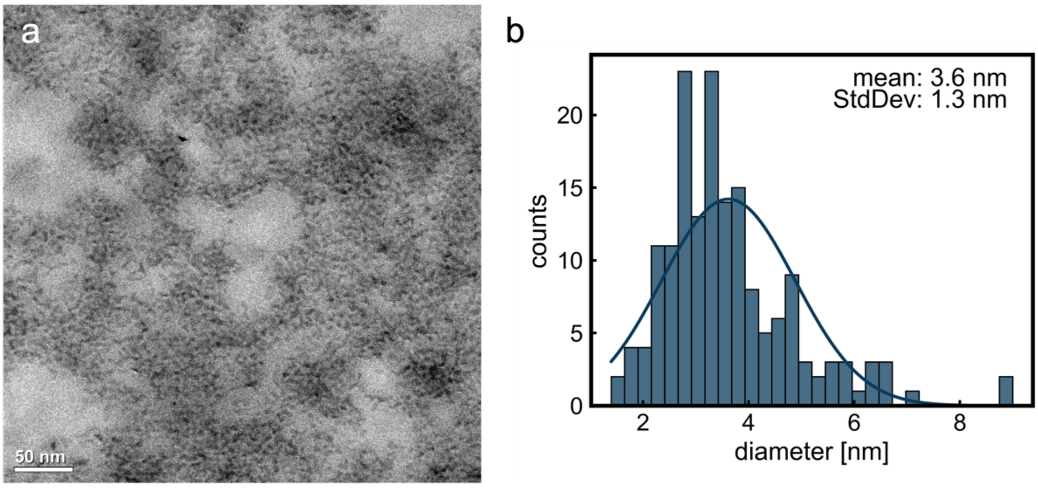

Figure SI2: **(a)** TEM image and **(b)** the particle size distribution of ZnS NPs as a final product of the described *in situ* synthesis (150 °C for 60 min) reveals an average size of 3.6 nm with a standard deviation of 1.3 nm.

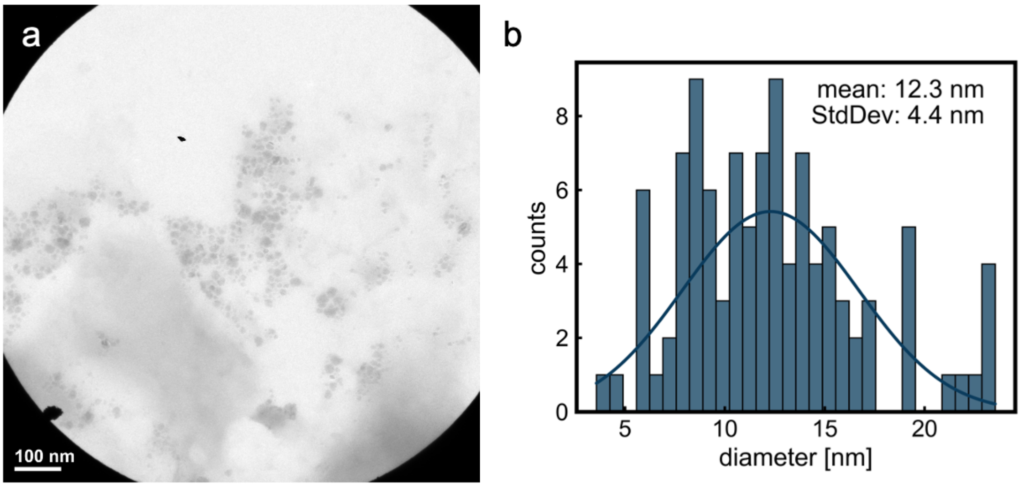

Figure SI3: HRTEM **(a)** and size analysis **(b)** of ZnS NPs as a final product of the described *in situ* synthesis (150 °C for 60 min) reveals an average size of 12.3 nm with a standard deviation of 4.4 nm. Therefore, the final product contains small and larger particles. The size distribution depends on the position of the grid.

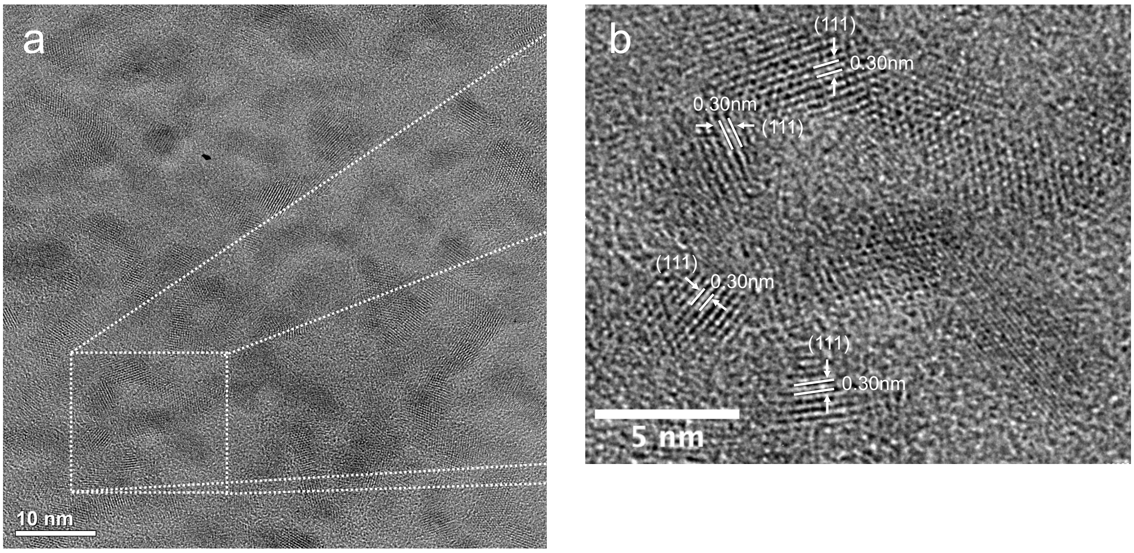

Figure SI4: HR-TEM analysis of ZnS NPs as a final product of the described *in situ* synthesis (150 °C for 60 min) shows 3-4 nm large particles.

DFT calculations of Zn^{2+} complexes in water/toluene:

All the structural optimization (left) and TD-DFT for XANES calculations (right), were performed with the following example ORCA .inp parameters:

```
!RKS B3LYP TightOpt TightSCF ZORA-def2-TZVP
ZORA
!FREQ Largeprint Printbasis
%maxcore 4000
%xes
CoreOrb 0
OrbOp 0
end
* xyz 2 1
…
```

```
!RKS B3LYP TightSCF ZORA-def2-TZVP D3BJ
RIJCOSX ZORA
!Normalprint MOREAD
%maxcore 4000
%moinp "Zn6H20_XES.gbw"
%tddft
orbwin[0]= 0,0,-1,-1 doquad true
nroots 300
maxdim 50
end

* xyz 2 1
…
```

The resulting output files are processed with orca_mapspc to export the spectra:
XAS: orca_mapspc name.mpi8.out absq -eV -x09000 -x110000 -n10001 -kw0.03

The .inp Files, the optimized .xyz files of all shown complexes, and the experimental raw data are available here: 10.25592/uhhfdm.16646

To propose the coordination of Zn^{2+} ions in the water and toluene phases, DFT calculations using ORCA were performed on many possible coordinations, shown in **Figure SI5**. The position and intensity of the white line were best reproduced with $[Zn(H_2O)_6]^{2+}$ in water and $[Zn(OA)_6]^{2+}$ in the toluene phase, respectively. The calculation of $Zn(Ac)_2$ was performed by cutting out a $[Zn(Ac)_4]^{2-}$ molecule out of the .cif file. All calculated spectra are redshifted by 18.3 eV to correct the mismatch between the calculations and experimental data.

MCR-ALS:

The MCR-ALS method use a constrained Alternating Least Squares (ALS) algorithm to solve a bilinear model. The bilinear equation is described as follows:

$$\mathbf{D} = \mathbf{C}\mathbf{S}^{\mathbf{T}} + \mathbf{E} \quad (1)$$

where **D** refers to the raw data set, **C** to the concentration profiles for each recovered compound, $\mathbf{S}^{\mathbf{T}}$ to the related pure spectra, and **E** to the matrix of residuals not accommodated by the model. The ALS algorithm operates based on the iterative principle of alternately fixing one set of variables while solving for the other. To elaborate, the process initiates by initializing $\mathbf{S}^{\mathbf{T}}$ and solving a linear algebra problem for **C** while maintaining $\mathbf{S}^{\mathbf{T}}$ as a constant. Subsequently, **C** is held steady, and the system is solved for $\mathbf{S}^{\mathbf{T}}$. This alternating cycle continues until convergence is attained. Convergence is defined by the criterion that, in two consecutive iterative cycles, the relative differences in the standard deviation of residuals between experimental and ALS-calculated data values fall below a predetermined threshold. The evaluation of the fit's quality, upon convergence, is based on the unexplained residuals **E**, signifying the disparity between the experimental data and the bilinear model. The **lack of fit (%)** is quantified as:

$$\textbf{lack of fit (\%)} = 100\sqrt{\frac{\sum_{i,j} e_{ij}^2}{\sum_{i,j} d_{ij}^2}} \quad (2)$$

Here, d represents an element within the data matrix, and e corresponds to the respective element in the residuals matrix **E**. Additionally, the model's explained variance $\mathbf{R}^2$ can be estimated using:

$$\mathbf{R}^2 = \frac{\sum_{i,j} d_{ij}^2 - \sum_{i,j} e_{ij}^2}{\sum_{i,j} d_{ij}^2} \quad (3)$$

The standard deviation of the residuals $\boldsymbol{\sigma}$ is given by:

$$\boldsymbol{\sigma} = \sqrt{\frac{\sum_{i,j} e_{ij}^2}{nm}} \quad (4)$$

In this context, n and m represent the dimensions of the raw data matrix, denoted as **D**. Additionally, the ALS algorithm can conform to physically or chemically meaningful constraints, such as enforcing non-negativity or unimodality for concentration and spectra profiles and closure.[1]

For the MCR-ALS analysis, the XANES data were structured into a matrix, where each row corresponds to an absorption spectrum recorded *in situ*. The number of rows is equivalent to the quantity of acquired spectra, and the number of columns corresponds to the number of data points in each spectrum. Before implementing the ALS optimization, the number of components and initial spectra was determined. The selection of the number of components was guided by the Singular Value Decomposition (SVD) outcomes. The computed eigenvalues from the data offer insights into the extent of variance each factor or component can elucidate. The factor associated with the highest eigenvalue represents the greatest variance, followed by the one with the second highest eigenvalue, and so forth. Eigenvalues below a certain threshold and with linear alignment indicate noise in the data. The determined eigenvalues in both datasets are illustrated in the Scree plot in **Figure SI5** and are listed in **Table SI1**.

To discriminate between noise and data in eigenvalues, one needs to check the correlation of eigenvalues, and check if they are linearly aligned. To underline their correlation, we implemented a new variable ∂_n, which describes the difference between the eigenvalue of component n and the next.

$$\partial_n = \frac{Eigenvalue\ of\ component\ n}{Eigenvalue\ of\ component\ n{+}1}$$

If $\partial_n \approx \partial_{n+1}$, the eigenvalues align linearly, and the component n corresponds to noise.

If $\partial_n \gg \partial_{n+1}$, the eigenvalues do not align linearly, and the component n corresponds to a considerable component.

In the MCR-ALS analysis of the water toluene interface (**Figure SI5a**), two compounds were used, based on a huge discrepancy between ∂_2 = 1917.23 % and ∂_3 = 9.56 % and a small discrepancy between ∂_3 = 9.56 % and ∂_4 = 34.28 %. Furthermore, an MCR-ALS analysis with three components does not achieve convergence and recover unphysical spectra.

In the MCR-ALS analysis of the *in situ* dataset (**Figure SI5b**), four components were used, based on a huge discrepancy between ∂_4 = 91.03% and ∂_5 = 24.80 %, and a small difference between ∂_5 = 24.80 %, and ∂_6 = 20.77 %. Furthermore, an MCR-ALS analysis with five compounds does not achieve convergence and recovers unphysical spectra. An MCR-ALS analysis with three compounds achieved convergence, but showed a meaningless concentration profile, as discussed in Figure SI6, and a larger difference between the recovered starting point and the first experimental spectra of the *in situ* run, as discussed in **Figure SI7**.

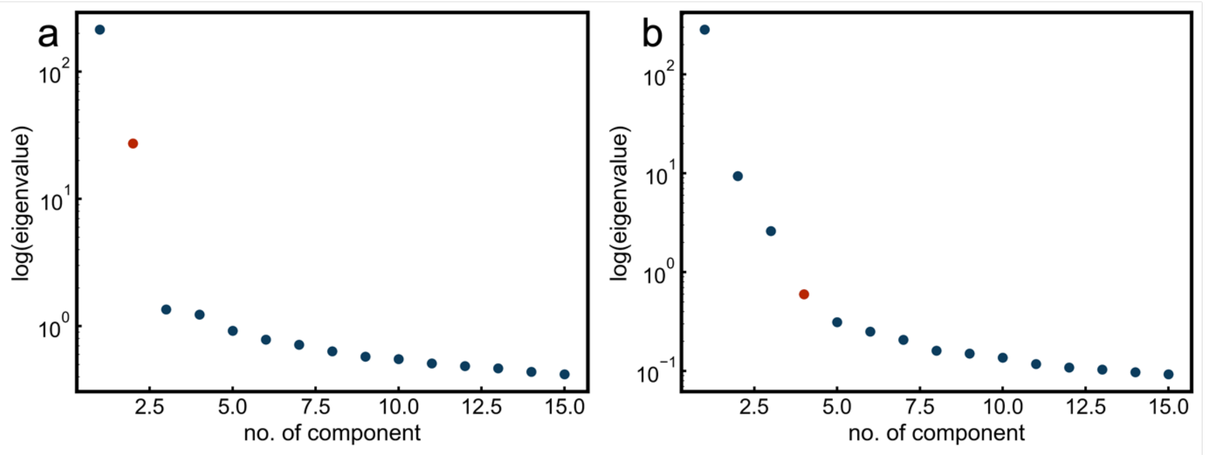

Figure SI5: Logarithmic scree plot of eigenvalues of **(a)** the scan across the water-toluene interface prior to the reaction and **(b)** the *in situ* preparation of ZnS NP. The number of used components is indicated in red. For the water-toluene interface scan, the eigenvalues suggest a usage of two components while for the *in situ* XAS in the toluene phase, four components were suggested by the behavior of the eigenvalues.

Table SI1: Eigenvalues determined by SVD of the MCR-ALS analysis.

Number of components	Eigenvalues of scanning across the water-toluene interface prior the reaction (Figure 2) (∂ to next eigenvalue)	Eigenvalues of synthesis of ZnS NP (Figure 3) (∂ to next eigenvalue)
**1**	214.056 (684.71%)	283.575 (2933.76%)
**2**	27.255 (1917.23%)	9.347 (260.11%)
**3**	1.352 (9.56%)	2.595 (335.40%)
**4**	1.234 (34.28%)	0.596 (91.03 %)
**5**	0.919 (17.37%)	0.312 (24.80%)
**6**	0.783 (9.82%)	0.250 (20.77%)
**7**	0.713	0.207

Table SI2: Fitting parameters of the MCR-ALS analysis.

	Fitting parameters of scanning across the water-toluene interface prior the reaction (Figure 2)	Fitting parameters of the synthesis of ZnS NP (Figure 3)
**fitting error (%)**	0.81	0.042
R^2	99.9934	100
**σ**	0.0095	0.00041

To verify the presence of four components in the *in situ* XAS run, we performed an MCR-ALS analysis with three components, which show an unphysical meaning of the concentration profile, as shown in **Figure SI6**, and larger discrepancies between the experimental starting point of the reaction, and the first recovered component, as shown in **Figure SI7**.

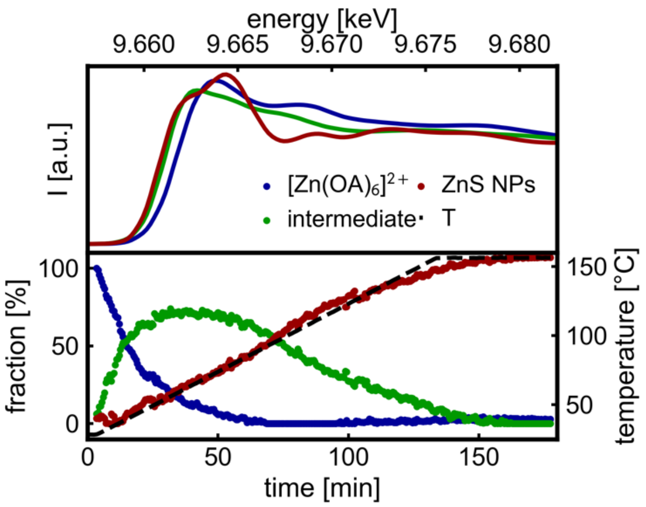

Figure SI6: MCR-ALS analysis of *in situ* synthesis of ZnS NPs in the toluene phase, using three components. The concentration profile of the ZnS NPs implies their formation already starting at 50 °C. Total scattering experiments do not provide evidence for such a low-temperature formation of ZnS.

Furthermore, the spectral features of the starting point and the final product were also slightly better reproduced using four components instead of three, as indicated by the standard deviations in the following **Figure SI7**.

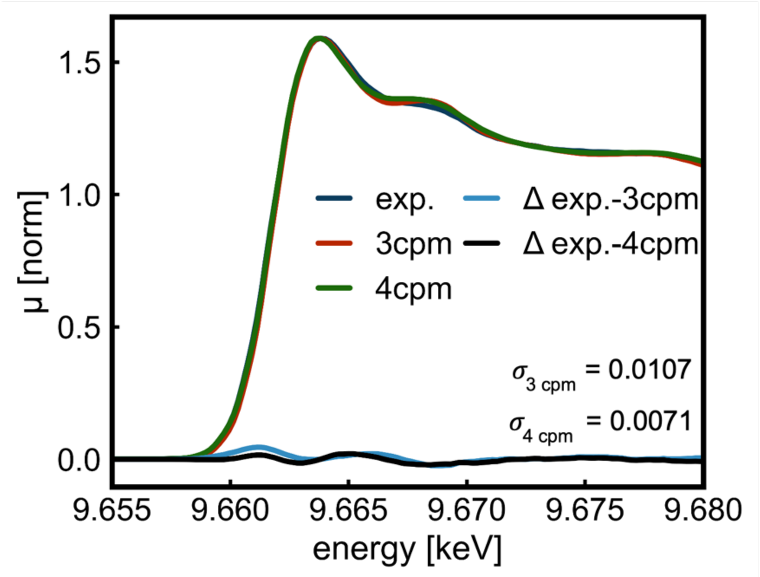

Figure SI7: Experimental spectra (blue lines) of the starting point compared with the MCR-ALS recovered contributions of three component (red lines) and four component (green lines) MCR-ALS analysis. The standard deviation between four component spectra is lower compared to three component spectra.

Beam damage study in water and toluene phase:

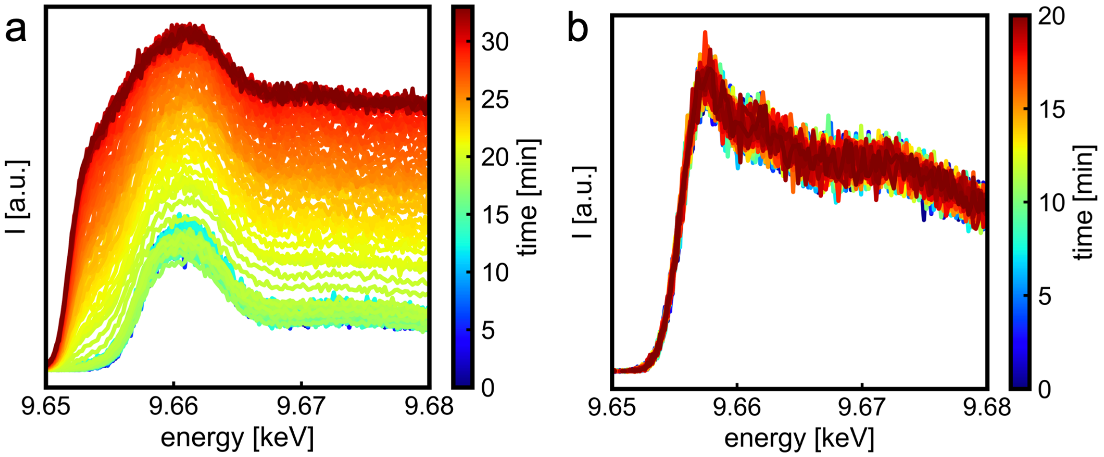

Figure SI8: Beam damage study of the precursor solution in **(a)** the water and **(b)** the toluene phase. In the water phase **(a)**, a deposition of metallic Zn on the reaction container could be observed, starting after 10 minutes of illumination. This was indicated by a shift of E_0 towards lower energies combined with an overall increased intensity. In the toluene phase, no formation of metallic Zn can be observed. The acquisition time for each spectrum was 15 s. The datasets were collected at Beamline ID24.

***In situ* diffusion of Zn^{2+} through the water-toluene interface:**

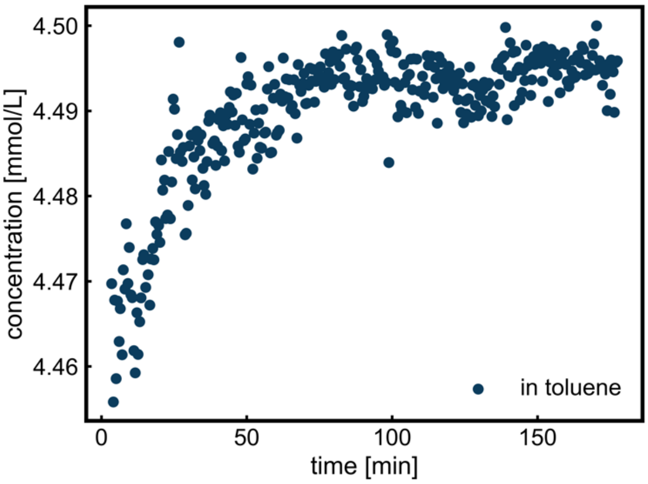

Figure SI9: Concentration profile of Zn^{2+} ions in the toluene phase during the *in situ* reaction. The Zn^{2+} concentration in the toluene phase reached its equilibrium after around 60 min reaction time (80 °C).

DFT calculations of Zn^{2+} complexes in water/toluene:

The E_0 of XANES spectra provides insights into the chemical environment and coordination of the absorbing atom. When the coordination environment of Zn^{2+} ions changes from nitrogen atoms to sulfur atoms, the E_0 of the XANES spectrum tends to shift to lower energy, as also indicated by DFT calculations, shown in **Figure SI10**. This shift can be explained by considering the electronic and structural differences between nitrogen and sulfur as coordinating ligands.

Firstly, sulfur is less electronegative than nitrogen, leading to a decrease in the effective nuclear charge experienced by the Zn^{2+} ion when coordinated by sulfur. This results in a higher degree of electron density or more covalent character at the zinc site, effectively reducing the ionization energy required to remove an electron from the zinc ion. Consequently, the E_0 value in the XANES spectrum is observed at a lower energy when Zn^{2+} is coordinated by sulfur atoms.

Additionally, sulfur's larger atomic radius compared to nitrogen can lead to an increase in the Zn-S bond distance relative to Zn-N, which also contributes to the observed lowering of the absorption edge energy. This increased bond distance can cause a reduction in the overall ligand field strength around the zinc ion, again contributing to a lower energy requirement for electron excitation in the XANES spectrum.

Thus, the shift of E_0 to lower energy in XANES upon changing the coordination from nitrogen to sulfur atoms reflects the combination of reduced effective nuclear charge and altered ligand field effects due to the differing electronic properties of sulfur compared to nitrogen.

To emphasize this point, we performed DFT calculations of tetrahedral and octahedral Zn-N and Zn-S coordination, as shown in **Figure SI10**. A general shift of the E_0 to lower energy from Zn-N to Zn-S coordination is observable.

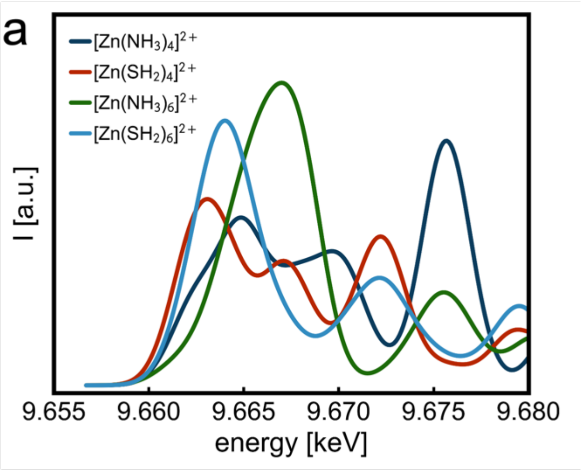

Figure SI10: DFT calculations of $[Zn(NH_3)_4]^{2+}$, $[Zn(NH_3)_6]^{2+}$ compared with $[Zn(SH_2)_4]^{2+}$ and $[Zn(SH_2)_6]^{2+}$.

The changes in the experimental HERFD-XANES data suggest the formation of intermediate structures, which suggest a component analysis, like MCR-ALS, as shown by comparing the experimental HERFD-XANES data of the reaction starting point at RT (blue line), at 60°C (green line) and at the end of the reaction (red line, ZnS NPs) in **Figure SI11.**

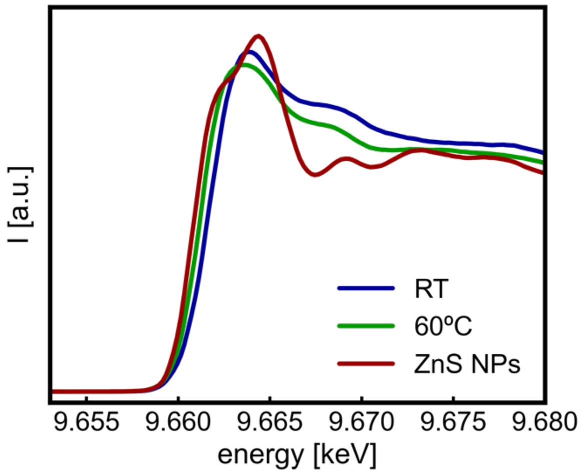

Figure SI11: Comparing experimental HERFD-XANES spectra of the reaction solution in toluene at room temperature, at 60°C, with the experimental HERFD-XANES.

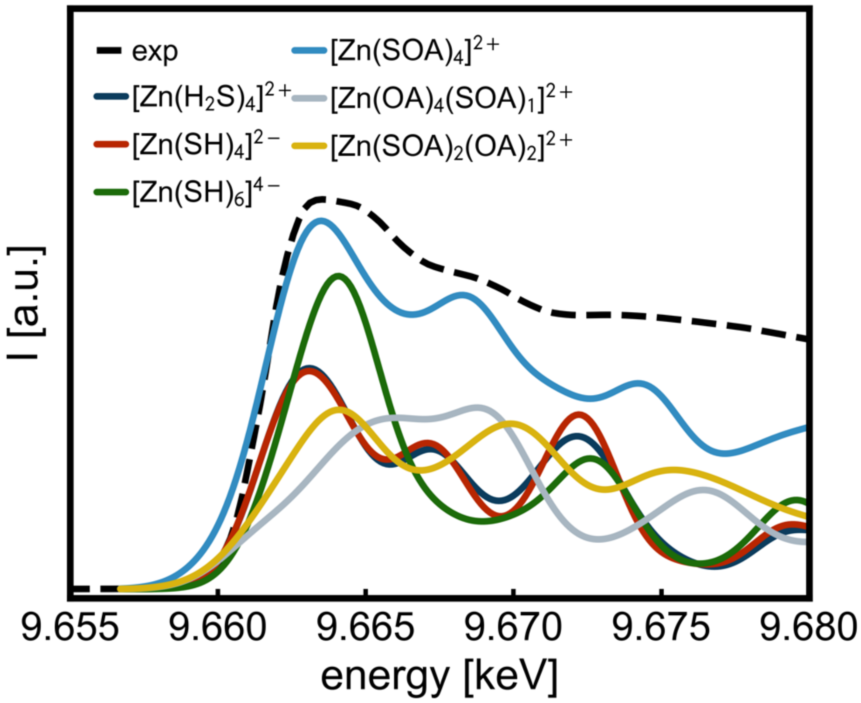

Figure SI12: The first recovered intermediate (black, dashed) compared with DFT calculations of $[Zn(H_2S)_4]^{2+}$, $[Zn(SH)_4]^{2-}$, $[Zn(SH)_6]^{4-}$, $[Zn(SOA)_4]^{2+}$, $[Zn(OA)_4(SOA)_1]^{2+}$ and $[Zn(SOA)_2(OA)_2]^{2+}$. None of the calculations explain the broad behavior of the white line, while the best agreement is achieved by assuming a $[Zn(SOA)_4]^{2+}$ complex.

Due to the differences between experimental and simulated data, we propose the presence of a $[Zn(SR)_4]^{2+}$ as the first intermediate, where the identity of the R is still unknown.

FDMNES calculations of ZnS nuclei and ZnS NPs:

The spectral shape of the final product of the *in situ* formation of ZnS NPs (red line) is compared with the ZnS nuclei (yellow line) and a ZnS reference (black dashed line), shown in **Figure SI13** (left). The ZnS NPs exhibit good agreement with the ZnS reference, while the ZnS nuclei differ from both, displaying less pronounced post-edge features and a weaker white line intensity. To identify the phase of the ZnS NPs, we performed FDMNES calculations of wurtzite ZnS (w-ZnS, blue line) and sphalerite ZnS (s-ZnS, gray line) in **Figure SI10** (right). The spectral overlap of ZnS NPs with s-ZnS confirms that the ZnS NPs are present in the sphalerite phase. Rietveld analysis of the commercial ZnS reference reveals a ZnS phase content of around 98% sphalerite and 2% wurtzite, as shown in **Figure SI14**.

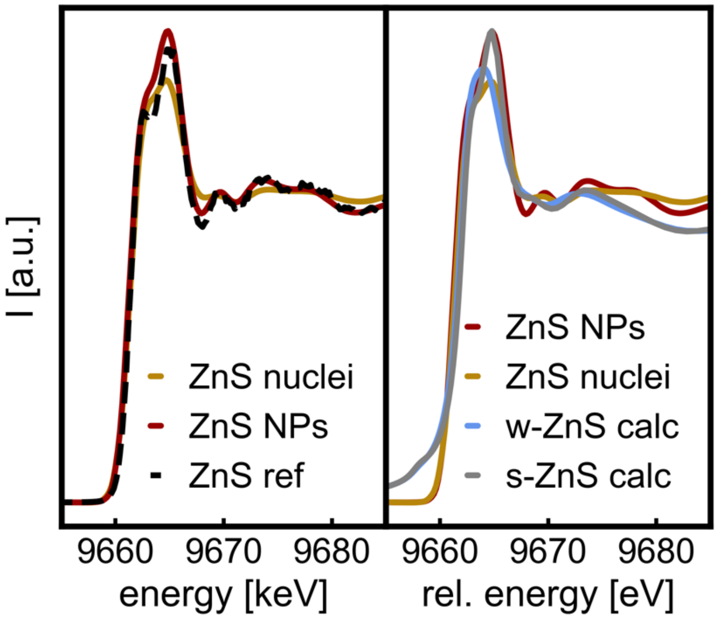

Figure SI13: (a) Experimental spectra of ZnS nuclei (yellow line) and ZnS NPs (red line) recovered by MCR-ALS analysis, compared with experimental spectra of a ZnS reference (black dashed line, Sigma Aldrich, Product 244627) (left). Experimental spectra of ZnS NPs (red line) compared to simulated spectra of wurtzite (yellow line) and sphalerite (red line) phases by FDMNES calculations (right).

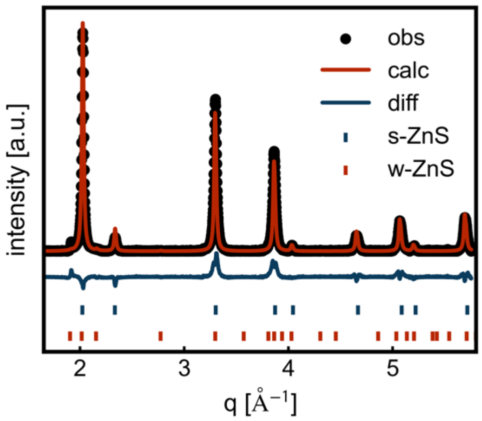

Figure SI14: Rietveld analysis of the ZnS reference shown in Figure SI10 (Sigma Aldrich, Product 244627) reveals a phase mixture of around 98% sphalerite and 2% wurtzite."

FDMNES calculation parameters:

The FDMNES calculations of w-ZnS and s-ZnS were performed with the following input parameters and the FDMNES code.

```
! Fdmnes indata file
! Calculation for the ZnS K-edge of ZnS
! Finite difference method calculation with convolution
Energpho
 Range                    ! Energy range of calculation (eV)
 -30. 0.2  5. 0.5 20. 1. 100.   ! first energy, step, intermediary
energy, step ..., last energy
 Z_Absorber
 30
 Edge
 K
 Radius
 5.0
Density
SCF
Spacegroup

Convolution


End
```

PDF analysis and the parameters of fit:

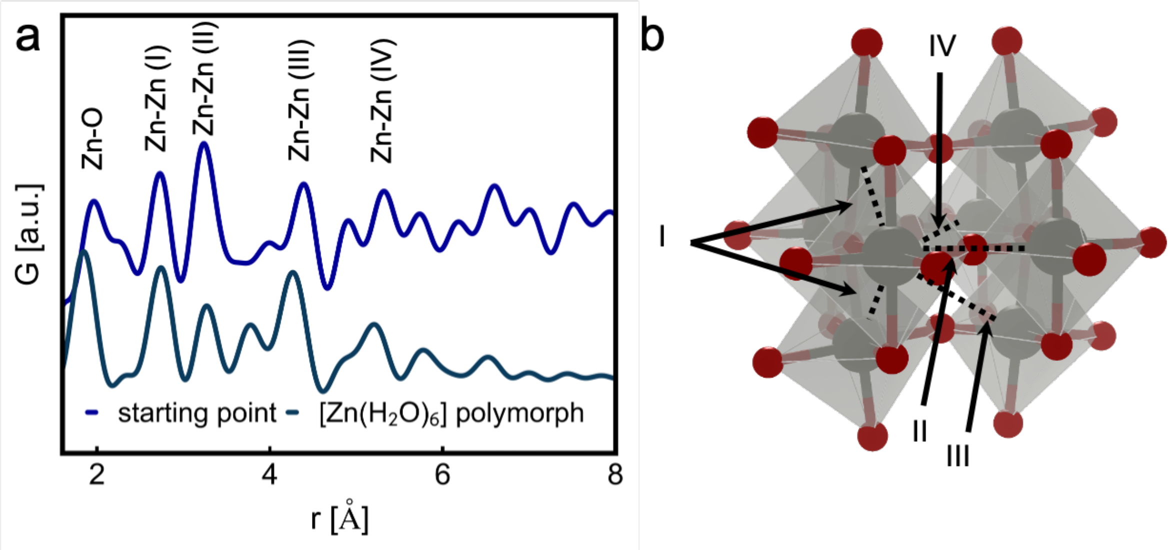

Figure SI15: (a) experimental PDF of the starting point (top) compared with simulated PDF of a $[Zn(H_2O)_6]^{2+}$ **(b)** Visualization of a $[Zn(H_2O)_6]^{2+}$ polymorph, where the Zn atoms are shown in gray and the oxygen is shown in red. The dashed lines correspond to Zn-Zn interatomic distances. The Index of the Zn-Zn interatomic distances (I, II, III, IV) corresponds to maxima in the PDF data, shown in (a). The hydrogen atoms are not shown in the scheme.

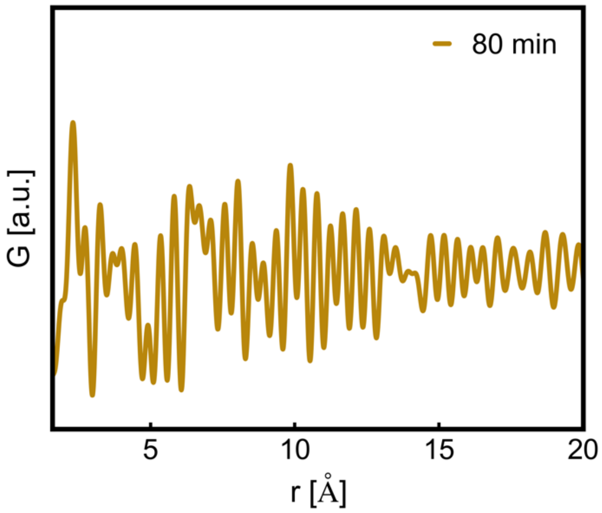

Figure SI16: experimental PDF after 80 minutes of reaction time, corresponding to the ZnS nuclei. The length of correlation is approximately 10 Å; beyond this value, the pattern primarily displays Fourier noise.

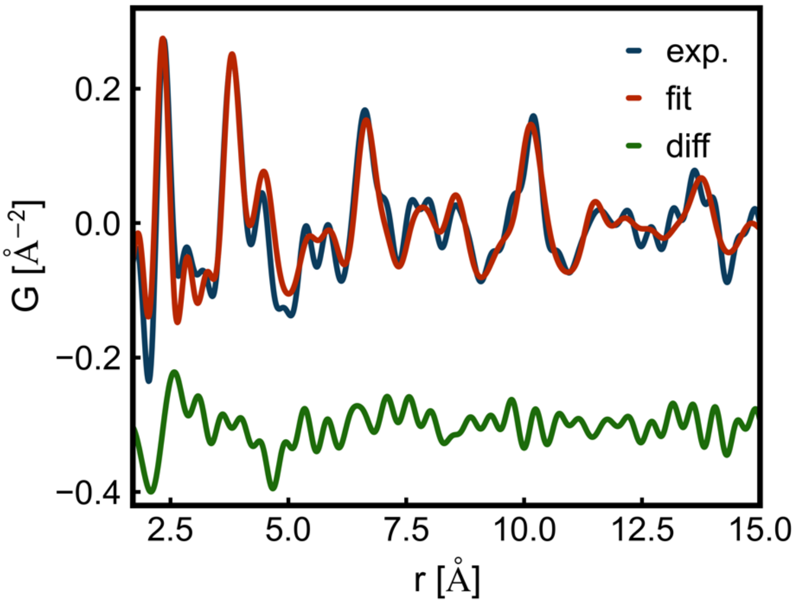

Figure SI17: PDF refinement of the final product with ZnS sphalerite. The goodness of the fit (Rw) is 0.37, while the average crystallite size was refined to be 2.4 nm.

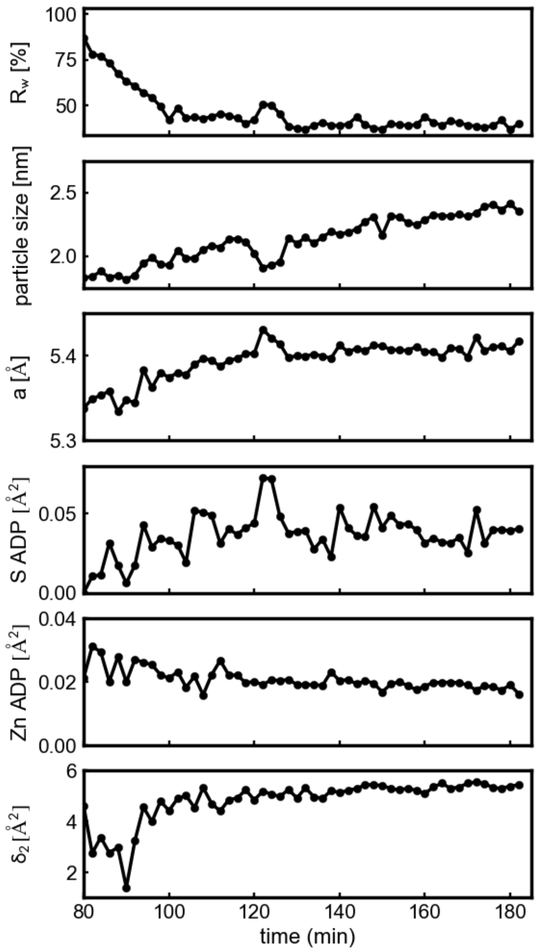

Figure SI18: Fitting parameters of *in s* PDF refinement during the synthesis of ZnS NP. The fit became reliable only after 100 min.

To underline the impact of the energy resolution on the experimental spectra, we compared in the following **Figure SI19** with experimental data of a ZnS reference (Sigma Aldrich Product 244627) measured in HERFD-XAS geometry at Beamline ID26 (ESRF) measured in standard transmission geometry at Beamline BM23 (ESRF).

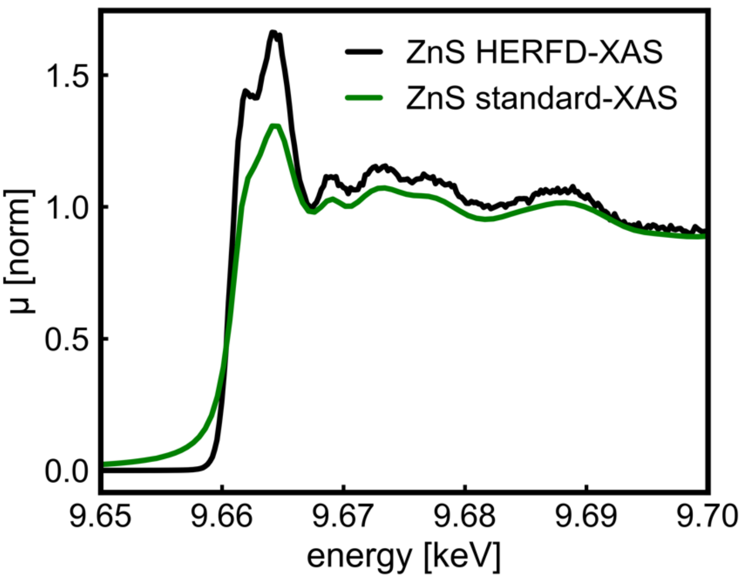

Figure SI19: Comparing XANES spectra of ZnS reference (Sigma Aldrich, Product 244627) measured in HERFD-XAS and standard transmission geometry. The HERFD-XAS spectra were acquired at beamline ID26, with a 90s acquisition time and a 0.1 eV step size. The transmission data were acquired at beamline BM23, with a 63-second acquisition time and a 1 eV step size. Bot samples were diluted with h-BN (ratio 1:10) and pressed into a pellet.

References:

(1) Jaumot, J.; Gargallo, R.; de Juan, A.; Tauler, R. A graphical user-friendly interface for MCR-ALS: a new tool for multivariate curve resolution in MATLAB. *Chemometrics and Intelligent Laboratory Systems* **2005**, *76* (1), 101-110. DOI: 10.1016/j.chemolab.2004.12.007.
